# Prejudice, Does It Exist or Not? Consumer Price Discrimination in Minority Entrepreneurship

**DOI:** 10.3389/fpsyg.2020.02180

**Published:** 2020-10-20

**Authors:** Feng Liu, Xin Liao, Cuiqin Ming

**Affiliations:** School of Public Affairs and Law, Southwest Jiaotong University, Chengdu, China

**Keywords:** minority entrepreneurship, consumer discrimination, ethnic discrimination, product quality, product price

## Abstract

Many prior studies on minority entrepreneurship have found that some consumers display a strong bias against products from minority ventures. Not surprisingly, discrimination against products sold by minority-owned businesses increases the failure rate for such ventures. This paper seeks to verify the extent of consumer discrimination for minority products, and investigates whether it varies among different products. Building on insights from the theory of consumer discrimination, we conducted a comparative behavior experiment on 155 subjects for the expected pricing of two new products (common products and products with ethnic characteristics). Consistent with prior literature, we found that potential consumers held a bias against common products from minority ventures and offered a lower price. However, the theory of consumer discrimination could not be applied to the products with ethnic characteristics. Instead, potential consumers viewed ethnic characteristics products from minority ventures as being high quality and offered higher prices. This finding complements the theory of consumer discrimination and provides useful knowledge for minority entrepreneurs: minority entrepreneurs can employ price discrimination to strengthen the ethnic brand’s impression by integrating ethnic cultural features into new products.

## Introduction

Ethnic minority entrepreneurship has become a hot issue in economic development, which reflects deep-seated political, cultural, ethnic and social issues such as ethnic equity, ethnic development opportunities, resource possession and distribution, cultural conflict and integration, and ethnic conflicts and exchanges. Ethnic discrimination is a widespread phenomenon around the world (Goldberg et al., 1996; Harrell, 2000; Pager and Shepherd, 2008; Brondolo et al., 2011; MacDonald and Hasmath, 2019) and is especially reflected in the labor market (Carlsson and Rooth, 2007; Wood et al., 2009; Kaas and Manger, 2012; Zschirnt and Ruedin, 2016; Neumark, 2018). Minorities who face persistent barriers and discrimination in the job market often turn to business ownership as an alternative, embracing entrepreneurship as a survival strategy in a difficult labor market (Horton and De Jong, 1991; Light and Rosenstein, 1995; Clark and Drinkwater, 2000; Laouenan, 2017). Accordingly, minority entrepreneurship has attracted the attention of many scholars in the field of entrepreneurship research. Prior minority entrepreneurship research shows that minorities still face inequality in workplace power and status (Elliott and Smith, 2004; Leicht, 2008; Joshi and Knight, 2015; Neville et al., 2018), are still woefully underrepresented (Kollinger and Minniti, 2006; Puryear et al., 2008; Chatterji and Seamans, 2012; Zhu et al., 2014; Freeland and Keister, 2016), and are subjected to stereotypes (Fairchild, 2008; Zapata et al., 2016). Even ethnic minority groups who choose to start a business experience discrimination. Thus, discrimination serves as a significant obstacle and barrier for minority entrepreneurship (Gomolka, 1977; Bewaji et al., 2015; Carter et al., 2015).

In particular, it is found that consumer preference (Ogbolu et al., 2015; Kuppuswamy and Younkin, 2020) and financing barriers caused by lending discrimination are key factors encountered by many minority entrepreneurs (Ekanem and Wyer, 2007; Roomi et al., 2009; Gill and Biger, 2012; Sandhu et al., 2012; Bewaji et al., 2015; Howell, 2019), although studies on consumer price discrimination for minority entrepreneurship are limited. Only a handful of scholars, such as Younkin and Kuppuswamy (2019), have investigated consumers’ discrimination against minority businesses. However, is the narrative surrounding discrimination against minority businesses entirely true? Further, is this discrimination likely to vary from product to product? In this article, we build on previous research on consumer discrimination (Becker, 1957) to suggest that consumer price discrimination against different products offered by minority-owned businesses varies extensively. Our comparative study examines two types of products offered by minority-owned ventures.

Using two behavior experiments, we tested our hypotheses among 155 undergraduate students. Our experiment, carried out in Southwest Jiaotong University, demonstrates that potential consumers hold a bias against common products from minority entrepreneurial ventures and tend to offer lower prices for the products. On the other hand, potential consumers believe that products with ethnic characteristics that are produced by minority-owned ventures are of good quality, and they tend to offer higher prices. Thus, this study deepens our understanding of minority discrimination, showing that consumers expect low prices for common products while offer high prices for products with ethnic characteristics that are produced by minority businesses.

The outline of the paper is as follows. In the section “Introduction,” we raise questions and generate hypotheses around these questions. We explore the existing literature and discuss consumer discrimination in minority entrepreneurship. In the section “Literature Review,” we describe the samples and research experiments. In the section “Hypothesis Development,” results of the statistical tests are reported and interpreted. In the concluding section, theoretical and practical implications are discussed and directions for future researches are proposed.

## Literature Review

Minority entrepreneurs are often owners and managers simultaneously, and their group membership is related to a common cultural heritage or origin and are considered by members outside the group to have these characteristics (Yinger, 1985; Aldrich and Waldinger, 1990; Zhou, 2004). Most studies show that minority entrepreneurs have some demographic characteristics that are different from those of non-minority entrepreneurs (Bewaji et al., 2015). Specifically, minority entrepreneurs are often born in low- or middle-class families, and many are young (below the age of 30), possess college degrees, and are motivated by both social and economic goals (Gomolka, 1977; Hisrich and Brush, 1986; Brush et al., 2007). Furthermore, ethnic minority groups have higher rates of self-employment and business ownership (Bates, 2000; Fairlie, 2004a). Although they have different motivations in starting businesses and possess stronger entrepreneurial intentions than non-minority entrepreneurs (Zhou, 2004; Edelman et al., 2010), minority entrepreneurs face higher failure rates in entrepreneurial activities (Kollinger and Minniti, 2006; Robb and Fairlie, 2007; Sullivan, 2007).

### Barriers to Ethnic Minority Enterprise

Factors affecting low rates of successful entrepreneurship for minority entrepreneurs include business experience (Fairlie, 2004b; Scarborough and Zimmerer, 2005), level of education and household assets (Bates, 1997; Fairlie, 1999; Bewaji et al., 2015), parental self-employment (Hout and Rosen, 1999; Fairlie and Robb, 2007), and family structure (Singh et al., 2009). Furthermore, some literature reveals that discrimination plays a role (Johnson and Thomas, 2008; Bewaji et al., 2015).

Several studies confirm that minority-run businesses are at a disadvantage compared to majority-run businesses. Traditional financial institutions are reluctant to invest in ethnic minority businesses (Altinay and Altinay, 2008; Sepulveda et al., 2011). For example, minority entrepreneurs often face discrimination from commercial banks, suppliers and insurance companies (Bates, 1991; Robb, 2013) and are less likely to access loans from financial institutions (Blanchard et al., 2008; Bewaji et al., 2015). Minority entrepreneurs are not only often denied credit, but they also pay higher interest rates than non-minority entrepreneurs (Daniels, 2004). In addition, discrimination reduces the availability of start-up capital, contributing to the high failure rate of minority entrepreneurs (Johnson and Thomas, 2008). Overall, minorities tend to face more serious financial constraints due to discrimination, which leads to lower business performance (Coleman, 2004; Fairlie and Robb, 2008). In this study, we focus on discrimination from consumers, another important factor determining the fate of new, minority-run ventures.

### Theory of Consumer Discrimination

In the theoretical literature, Becker (1957) was the first to study discrimination in entrepreneurship among ethnic minorities. In his seminal work, Becker proposes the theory of consumer discrimination, which was also called the taste-based theory of discrimination, and argues that taste-based discrimination derives from aversion to cross-racial interaction. In other words, individuals may simply dislike a particular group and choose not to transact business with them. Furthermore, Becker (1971) proposes that cross-ethnic transactions cost more and may lead to treatment discrimination.

Based on this theory, Borjas and Bronars (1989) show that consumer discrimination contributes partly to the large observed variance in self-employment rates across racial groups (e.g., Asians, Hispanics, blacks, and whites). In addition, customer discrimination causes more discrimination in business transactions (Antecol and Cobbclark, 2008). For example, in the southern United States consumers discriminate against black service providers by tipping them less than white service providers (Lynn et al., 2008). It has been confirmed that discrimination against African Americans occurs on the job, partly as a result of consumer discrimination (Laouenan, 2017). Indeed, Becker’s theory is often used to explain reluctance to associate with people of different ethnicities (Broyles and Keen, 2010; Hekman et al., 2010). Prior studies also utilize it to explore the idea that consumers may expect minority entrepreneurs to reduce their prices (Avery et al., 2015; Younkin and Kuppuswamy, 2019). In the same vein, the theory of consumer discrimination would predict that consumers may discriminate against new products due to the products having different producers. Although such consumer discrimination has not been studied in depth in the Chinese market, prior studies have noted the importance of consumers’ influence on minority entrepreneurship.

### Minority Entrepreneurship Discrimination in China

China is a multiethnic nation, and over the past decade there has been a significant increase in minority entrepreneurship throughout the country. Ethnic minority entrepreneurship in China means that enterprises are owned and operated by ethnic minorities (Smallbone et al., 2010; Appiah et al., 2019; He et al., 2019). Due to data limitations and political sensitivities, however, research on ethnic minority entrepreneurship in China is limited (Howell, 2019).

Some scholars point out that differences in discrimination may occur between Han majority-based and minority-based enterprises. For example, studies by Hannum and Xie (1998) and Howell (2011) show that ethnic minorities are in an economically disadvantaged position due to the development of the labor market and social stratification. Many studies support this viewpoint, underlining that ethnic minorities experience economic discrimination (Zang, 2008; Howell and Fan, 2011; Howell, 2016, 2017). Specifically, Howell (2016) indicates that ethnic minorities are more vulnerable to liquidity shocks relative to the Han majority.

The study by Howell (2019), which is based on the first wave of the China Household Ethnic Survey, provides evidence from minority areas of China about the association between ethnicity and discrimination. In particular, it is found that minority-operated enterprises perform worse than Han-operated enterprises, and that the main reason is the difficulty that ethnic minorities have when obtaining external financing, thus suggesting underlying discrimination (He et al., 2019). As pointed out by Howell (2019), the identification of discrimination is only the first step. Further research that analyzes the underlying causes of discrimination and confirms whether ethnic-based differences are due to lending discrimination, selection effects, or both, are greatly needed. Aside from financing discrimination, it is still unclear whether other forms of discrimination exist, including consumer discrimination against new products from minority entrepreneurs. The purpose for this study is to provide evidence for whether the consumer price discrimination in minority entrepreneurship exists.

## Hypothesis Development

Consumer behaviors are influenced by personal experiences, perceptions of legitimacy, and cultural attitudes and stereotypes (Greenwald and Banaji, 1995). It is generally accepted that consumers’ business decisions are closely related to their trust in a business establishment (Child and Mollering, 2003; Gounaris, 2005). Ogbolu et al. (2015) argue that consumers have different perceptions of minority and non-minority entrepreneurs, which can affect patronage of firms. For example, attitudes from consumers affect black entrepreneurs whose businesses may suffer due to the effects of negative stereotypes held about African Americans (Fairchild, 2008). Most employers across industries hold that the locus of bias exists not within the organization, but rather, comes from the consumers who patronize the organization (Kuppuswamy and Younkin, 2020). Consumers tend to be cautious about what and from whom they buy, and are often concerned about being exploited by new or unfamiliar businesses (Ogbolu et al., 2015). In some cases, cross-racial interactions between consumers and minorities may make customers feel uncomfortable. Minorities face prejudice based on their ethnic background, which can negatively affect their businesses (Rogoff and Heck, 2008). Furthermore, some minorities seem to internalize the negative stereotypes and prejudices, which may lead to further economic and social disadvantages (Sarnoff, 1960; Fairchild, 2008; Zapata et al., 2016).

Consistent with this view, empirical research in China provides some evidence that minorities are more likely to fall into poverty due to their disadvantages (Gustafsson and Sai, 2009). The Han majority, similar to whites in the United States, dominate the economic, political, and cultural spheres (Blum, 2001), and many view themselves as more advanced than minorities (Harrell, 1995; Maurer-Fazio et al., 2007). Such prejudices may result in unfavorable attitudes toward minority entrepreneurs.

**FIGURE 1:**
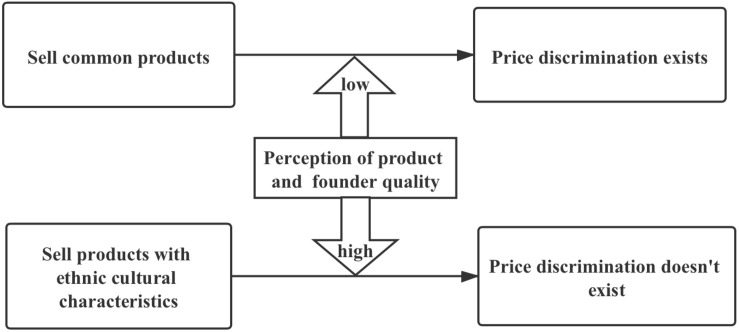
Theoretical process model.

The theory of consumer discrimination suggests that consumers discriminate against minority entrepreneurs and expect that minority entrepreneurs will reduce their prices (Becker, 1957; Avery et al., 2015; Younkin and Kuppuswamy, 2019). Consequently, we propose that consumers expect the prices of minority entrepreneurs’ products to be lower than Han entrepreneurs’ products when the two groups are selling common products at the same time. Hence, based on the previous arguments, as depicted in Figure 1, this paper proposes the following hypothesis:

**Hypothesis 1:** Consumers expect minority entrepreneurs to sell common products at lower prices than products sold by Han majority entrepreneurs.

We offer two explanations for why consumers may expect lower prices on common products sold by minority entrepreneurs. First, under taste-based discrimination (Becker, 1957), consumers who dislike a particular group and get utility from engaging in discrimination are willing to give up other priorities so as to satisfy their discrimination. Second, the expectation of lower prices may be due to statistical discrimination (Phelps, 1972; Arrow, 1973), which originates from information asymmetry. It is suggested that potential consumers may consider a particular group to be unproductive or less productive than other groups. That is, the consumer’s discrimination may derive from unobserved variables (i.e., statistical discriminants) whereas potential consumers are likely to use entrepreneurial ethnic identity to infer lower observable quality of entrepreneurs and products (Younkin and Kuppuswamy, 2019). Therefore, consumers may apply statistical discrimination to compensate for the incomplete information about minority entrepreneurs’ productivity and quality.

In particular, an empirical study finds that race influences the number of tips that servers get from customers (Lynn et al., 2008). In addition, discrimination in the consumer market depends on the racial composition of the local community (Antecol and Cobbclark, 2008). Similarly, scholars such as Ogbolu et al. (2015) further confirm that consumers’ perceptions of a founder’s race affect their intentions of patronizing that entrepreneur’s firm, which is interpreted as evidence that consumers utilize race and ethnicity to make their decisions. Therefore, we propose that, in cases where minority entrepreneurs and Han entrepreneurs sell common products simultaneously, consumers will assess the qualities of products according to the entrepreneur’s ethnicity.

Hence, based on the abovementioned arguments, we propose the following hypothesis:

**Hypothesis 2:** When assessing common products, consumers will rate the quality of products lower for minority entrepreneurs than Han majority entrepreneurs.

In previous paragraphs we explored the phenomenon of consumer discrimination against minority entrepreneurs for common products. Next, we analyze whether these consumer discriminations may be different due to the difference in products. Consumers may hold different attitudes toward ethnic products endowed with cultural elements, and many may believe that minority entrepreneurs who are familiar with such cultural elements will produce better products than Han-majority entrepreneurs.

A wealth of evidence illustrates that culture plays an important role in the process of economic development (Parker, 1997; Minkov and Hofstede, 2012). Specifically, studies such as Ma and Todorovic (2012) show that culture may influence the characteristics of the entrepreneurship process, influencing local economic development. In the entrepreneurship process, culture functions as a guide with the entrepreneur as the catalyst (Lee and Peterson, 2000). In a similar vein, Rafiq (1992), Morrison (2000), and Thai and Turkina (2014) argue that culture is one of the major determinants of an individual’s attitudes toward entrepreneurship, and is vital force for entrepreneurial motivation in influencing consumer attitudes and creating demand for certain products and services. Consequently, we propose that consumers may not discriminate against ethnic products that are produced by minority entrepreneurs.

Hence, based on the abovementioned arguments, we propose the following hypothesis:

**Hypothesis 3:** Consumers will be less likely to hold product price discrimination against minority entrepreneurs when products have ethnic cultural characteristics.

For minority entrepreneurs, their group relationship is rooted in a common cultural heritage (Yinger, 1985; Aldrich and Waldinger, 1990; Zhou, 2004). To explain how minority entrepreneurs appeal to consumers, existing research shows that entrepreneurs have a strong connection with customers who fit their entrepreneurial concepts (Thébaud, 2010), and that they can gain consumer support by emphasizing their fitness in other empirical areas (Kacperczyk and Younkin, 2017). Hence, if minority entrepreneurs emphasize their products’ ethnic cultural characteristics, we would expect that consumers will propose that such products fit better than similar products produced by Han-majority entrepreneurs. Obviously, consumers will use entrepreneurs’ ethnicity to predict the qualities for ethnic products. Cultural distinctiveness can provide a big advantage for minority entrepreneurs by helping them to offset the potential negative attitude from consumers who dislike them for their ethnicity. Consequently, we propose that, under the same conditions, consumers may assume that products with ethnic cultural characteristics produced by minority entrepreneurs are of higher quality than products produced by Han entrepreneurs.

Hence, based on the prior arguments, we propose the following hypothesis:

**Hypothesis 4:** Consumers will assume that minority entrepreneurs sell products with ethnic cultural characteristics at higher prices than common products produced by Han majority entrepreneurs.

**Hypothesis 5:** Consumers will rate the quality higher for products with ethnic cultural characteristics that are produced by minority entrepreneurs compared to products produced by Han majority entrepreneurs.

## Methodology

Lab experiments are very popular in discrimination research and have been used to test for discriminatory decision making, as well as understanding the nature of discrimination and studying behaviors that may lead to discriminatory results (Baert and De Pauw, 2014; Dittrich et al., 2014; Reuben et al., 2014; Neumark, 2018). This study aims to clarify, mainly through the means of behavior experiment, whether the consumer price discrimination in minority entrepreneurship exists, and if so, under what circumstances. Therefore, we designed a two-stage experiment. The first stage of the experiment aims to conduct a study on price discrimination of common products. The second stage of the experiment is designed to examine price discrimination of products with ethnic cultural characteristics.

### Participants

One hundred and fifty-five paid participants in the subject pool of a Southwestern China university’s lab took part in this study. The participants are undergraduate students who were majoring in management. They were experiencing an innovative entrepreneurship education that is compatible with the socioeconomic development and education reform in China (Sun, 2020). They were told that the study helped entrepreneurs develop and decide a suitable product price. The 155 participants ranged in age from 18 to 25 years old (Mean = 19.46, SD = 0.94). In the sample, a total of 92.3% of the participants were Han majority. Respondents reported as majority female (61%). Participants were randomly assigned to one of three experimental conditions where disclosure of ethnic cultural elements and characteristics (vs. a control condition) was the manipulated factor.

### Procedure

**FIGURE 2:**
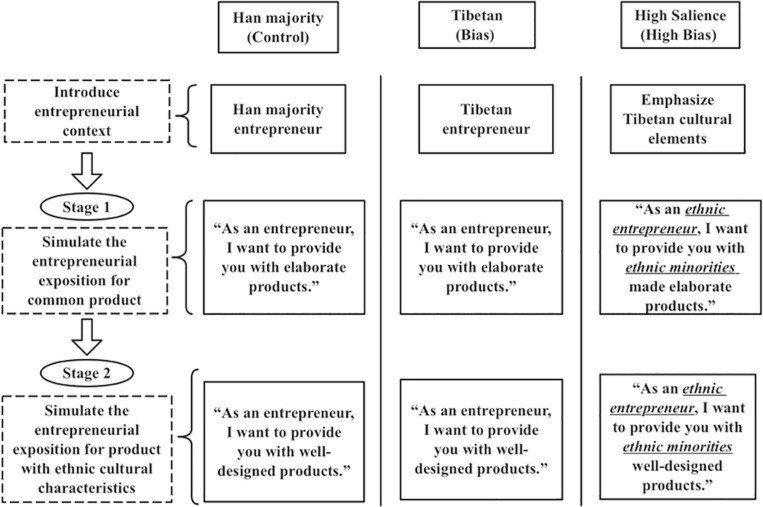
The flow of experiment.

The experiment process is shown in Figure 2. Prior to making their suggestion, participants were randomly assigned to one of three conditions: Han majority, Tibetan, and High Salience. This study selected the Tibetan as the context object because it is, culturally-speaking, the furthest from the Han majority and most easily identifiable as different (Hasmath, 2014). Those randomly assigned to the *Han majority condition* learned that they were shown entrepreneurship programs by a Han male entrepreneur. Participants assigned to the *Tibetan condition* were told that they were shown startup programs by a Tibetan male founder. Participants assigned to the *High Salience condition* were told that they were shown programs by a Tibetan male founder while emphasizing his ethnicity.

Next, researcher presented the participants two startup items on a large screen in the lab. During the first stage, participants were presented with the common product, that is, a thermos cup with a temperature display. In the *Han majority condition* or the *Tibetan condition*, participants were shown a monolog by a Han majority or Tibetan entrepreneur that “as an entrepreneur, I want to provide you with elaborate products.” In the *High Salience condition*, participants were shown a monolog by a Tibetan entrepreneur that “as an ethnic entrepreneur, I want to provide you with ethnic minorities made elaborate products.”

Then, at the second stage, participants were presented with the product with ethnic cultural characteristics, that is, a Tibetan silver bracelet. In the *Han majority condition* or the *Tibetan condition*, participants were shown a monolog by a Han majority or Tibetan entrepreneur that “as an entrepreneur, I want to provide you with well-designed products.” In the *High Salience condition*, participants were shown a monolog by a Tibetan entrepreneur that “as an ethnic entrepreneur, I want to provide you with ethnic minorities well-designed products.”

Finally, participants were asked what price (RMB) they would recommend to the entrepreneur after watching each of the entrepreneurship programs for at least three minutes. Finally, they were asked to complete and answer a series of questions, including their perception of product quality and founder quality, which are listed in our questionnaire.

### Measures

#### Perception of Product Quality

To assess the extent to which participants perceived product quality, participants completed the Perceived Quality Indicators Scale (Dodds et al., 1991). The 5-item scale was used to ask participants to evaluate the extent to which their perception of entrepreneurial products (e.g., “The likelihood that the product would be reliable is very high” and “The workmanship of product would be very high” and “The likelihood that this product is dependable is very high”) on a Likert-type scale ranging from 1 (disagree strongly) to 7 (agree strongly). Participants’ responses were averaged, with higher scores indicating much higher perception of product quality. The Perceived Quality Indicators demonstrated good reliability and validity (Cronbach’s α = 0.885, KMO = 0.815) for the current sample.

#### Perception of Founder Quality

To evaluate the extent to which participants perceived product quality, we used a version of the founder quality scale that included honesty, dependability, level of organization, confidence, and likeability (Younkin and Kuppuswamy, 2019). To test this, respondents were asked to assess the extent to which their perception of an entrepreneur (e.g., “From this product perspective, I think the entrepreneur is very honest” and “the entrepreneur is very dependable”) on a Likert-type scale ranging from 1 (disagree strongly) to 7 (agree strongly). The Perceived Founder Quality Scale showed good reliability and validity (Cronbach’s α = 0.847, KMO = 0.763) for the current sample.

## Analysis and Results

We used confirmatory factor analysis to prove that the Perceived Quality Indicators exhibit a good fit with χ2/df = 12.964, SRMR = 0.046, GFI = 0.924, CFI = 0.937, and NFI = 0.933 and the Perceived Founder Quality Scale presents a good fit with χ^2^/df = 18.383 and SRMR = 0.091.

Table 1 reports correlational and descriptive statistics for all variables. The correlations among the study variables are significant and less than 0.8, indicating that there are no multicollinearity relationships. It can thus be seen through the table of correlation results that the correlations of gender, age, ethnicity, perception of common product quality, perception of founder quality for common product, perception of product with ethnic characteristics quality, and perception of founder quality for products with ethnic characteristics are 1. Thus, the convergent validity of all variables is verified.

**TABLE 1:** Descriptive statistics and correlations.

Variables	Mean	SD	1	2	3	4	5	6	7
1. Gender (1 = male)	1.6129	0.48866	1.000						
2. Age	19.4581	0.94139	−0.318**	1.000					
3. Ethnicity (1 = Han majority)	1.0774	0.26812	0.082	0.064	1.000				
4. Perception of common product quality	5.3948	0.89034	0.040	−0.016	−0.205*	1.000			
5. Perception of founder quality for common product	4.9161	0.85215	0.074	0.100	−0.142	0.524**	1.000		
6. Perception of product with ethnic characteristics quality	5.1419	1.04172	−0.039	−0.053	−0.156	0.554**	0.501**	1.000	
7. Perception of founder quality for product with ethnic characteristics	4.8839	0.91136	−0.064	0.029	−0.144	0.363**	0.545**	0.744**	1.000

*p < 0.05 and **p < 0.01.

**FIGURE 3:**
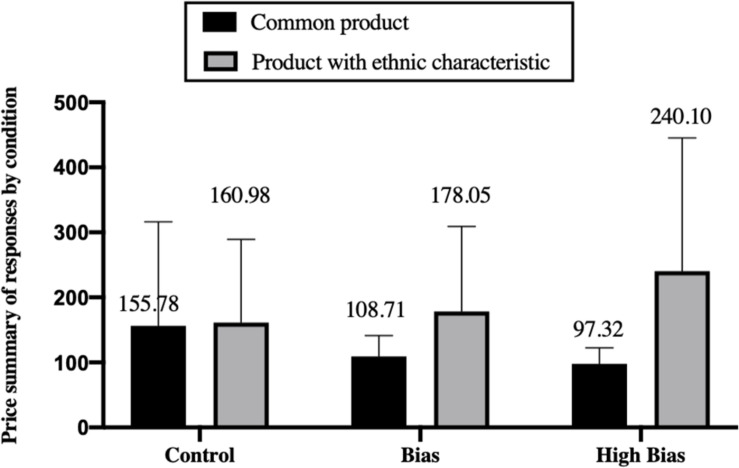
Price summary of responses by condition.

Price summaries of the respondent for each condition (control, bias, and high bias) are available in Figure 3. Preliminary results show that, for common products, prospective consumers would hold that the product price of a Han-majority entrepreneur (155.78) is higher than a minority entrepreneur (108.71) or ethnic entrepreneur who emphasizes their ethnic minority cultural elements (97.32). On the contrary, for common products with ethnic characteristics, prospective consumers would propose that the product price of an ethnic entrepreneur who emphasizes their minority cultural elements (240.10) is higher than a minority entrepreneur (178.05) or Han majority entrepreneur (160.98).

To further test the first hypothesis regarding price discrimination for common products, we use an unpaired Welch’s *t*-test and find that participants shown the bias condition (mean = 108.71, SD = 32.39) recommend a price to the founder that is significantly lower than those shown the control (mean = 155.78, SD = 160.08, *t* = 2.042, and *p* = 0.046), and that respondents shown the High Salience condition (mean = 97.32, SD = 24.83) recommend a price that is significantly lower than those shown the control (*t* = 2.552, *p* = 0.014). Based on the above analysis, hypothesis 1 is supported.

In models 1–3 of Table 2, by using Ordinary Least Squares regression, we model recommended price for common products as a function of participant characteristics. In model 3, we also find that participants in the bias condition recommend a lower price (β = −0.242, *p* = 0.010) than those in the control condition, and that price difference is statistically significant, which again proves hypothesis 1.

**TABLE 2:** The regression results of OLS estimates of recommended price.

	Stage 1 (common product)			Stage 2 (product with ethnic characteristic)		
	Model 1	Model 2	Model 3	Model 4	Model 5	Model 6
**Participant characteristics**						
Gender	0.104		0.065	−0.212*		−0.173*
Age	0.076		0.024	−0.217*		−0.185*
Ethnicity	−0.069		−0.093	−0.030		−0.019
**Condition**						
Bias		−0.234*	−0.242*		0.051	0.035
High Bias		−0.284**	−0.280**		0.232*	0.163
Constant	−37.134	155.776**	122.864	1042.365**	160.978**	887.258**
R2	0.015	0.069**	0.078*	0.066*	0.044*	0.085*

*p < 0.05, **p < 0.01, and N = 155.

Next, in order to test the second hypothesis that consumers will rate minority entrepreneurs as lower in quality than Han-majority entrepreneurs for common products, we use Welch’s *t*-test and find that participants rate the product of Han majority entrepreneurs (mean = 5.44, SD = 0.84) as more reliable than the products of Tibetan entrepreneurs (mean = 5.32, SD = 0.95, *t* = 0.706, and *p* = 0.48). Besides, the strongest effect indicates that participants rate the Han majority entrepreneur (mean = 5.03, SD = 0.93) as more trustworthy than the Tibetan entrepreneur (mean = 4.82, SD = 0.82, *t* = 1.226, and *p* = 0.223). Based on the above analysis, hypothesis 2 is not supported.

To further test the third and fourth hypotheses regarding no price discrimination for products with ethnic characteristics, we also used an unpaired Welch’s *t*-test and found that participants shown the *Han majority* condition (mean = 160.98, SD = 127.71) recommended a price that was significantly lower than those shown *the High Salience condition* (mean = 240.10, SD = 204.736, *t* = −2.318, and *p* = 0.023), and that respondents shown the *Han majority condition* recommend a price that is not significantly lower than those shown the bias (mean = 178.05, SD = 130.688, *t* = −0.676, and *p* = 0.501). In model 5 of Table 2, we find that participants in the High Bias condition recommend a higher price (β = 0.232, *p* = 0.013) than those in the *Han majority condition*. Based on the above analysis, hypotheses 3 and 4 are supported.

Finally, we test the fifth hypothesis that consumers will rate minority entrepreneurs as higher in quality than Han-majority entrepreneurs for products with ethnic cultural characteristics by using Welch’s *t*-test. We find that participants rate the product of high bias condition (mean = 5.46, SD = 0.86) as more reliable than the product of Han entrepreneurs (mean = 4.70, SD = 0.94, *t* = −4.224, and *p* = 0.000). Furthermore, the results of this analysis show that participants rate entrepreneurs in a high bias condition (mean = 5.14, SD = 0.80) as more dependable than Han entrepreneurs (mean = 4.52, SD = 0.93, *t* = −3.518, and *p* = 0.001). The results indicate that emphasizing ethnic cultural elements of products with ethnic characteristics is a good way for minority entrepreneurs to garner higher prices for their products. Based on the above analysis, hypothesis 5 is supported.

## Discussion

This article examines consumer price discrimination against minority entrepreneurs in China. The experimental data were collected from a famous university. From the results we may draw several conclusions. First, in accordance with hypothesis 1, this study reveals that prospective consumers assume that minority entrepreneurs sell common products at lower prices than Han majority entrepreneurs. Second, in contrast to hypothesis 2, we find no statistically significant differences between the Han majority condition and Tibetan condition. In other word, consumers who want the ethnic minority entrepreneur to adopt a lower price for common products did not indicate any expectation that minority entrepreneurs would be less capable of delivering a quality product. Those results comply with Younkin and Kuppuswamy (2019) proposition that prospective consumers anticipate lower prices for products from minority entrepreneurs. This conclusion is consistent with existing studies.

In addition, the result shows that the consumer price discrimination is no longer true for products with ethnic characteristics. Hypothesis 3 proposes that consumers will be less likely to hold product price discrimination against minority entrepreneurs when products have ethnic cultural characteristics. Analysis of the results indicates that participants shown the *Han majority condition* recommended prices that were not significantly lower than those shown the *Tibetan condition*. That is, minority entrepreneurs are less likely to face consumer price discrimination when selling products with ethnic cultural characteristics. Combining with hypothesis 4 and 5, the current study finds that prospective consumers will assume that the quality of ethnic products from minority entrepreneurs is higher and therefore offer a higher price. This finding overturns the conclusion of existing literature (Zang, 2008; Howell and Fan, 2011; Howell, 2016, 2017; Younkin and Kuppuswamy, 2019). Theoretically, culture plays an important role in the process of economic development and is one of the major determinants of individuals’ attitudes toward entrepreneurship (Rafiq, 1992; Parker, 1997; Morrison, 2000; Minkov and Hofstede, 2012; Thai and Turkina, 2014). Thus, price discrimination against minorities does exist in common products. However, this does not hold true for products with ethnic characteristics.

The findings of this study have theoretical and practical implications. A key implication for the current study is that minority entrepreneurs are advised to avoid highlighting the characteristics of ethnic minorities so as to avoid price discrimination if they choose a common product. Further, they should make use of price discrimination by strengthening the impression of ethnic brands, integrating ethnic culture into the products, and improving the premium ability of products if they choose the products with ethnic characteristics. Consequently, this study contributes to minority entrepreneurship by providing practical implications in the context of China.

## Limitations and Future Research Direction

Finally, this work has certain limitations, providing opportunities for future research. First, the sample size is small and homogeneous as all participants are from the same university. Future research is needed to explore the consumer discrimination against ethnic minorities with different identities. Second, although we found that potential consumers hold a bias against common products from minority ventures and offer a lower price, we did not find the underlying mechanism and reason. Besides, we can further explore other mechanisms of consumer price discrimination besides consumers’ perception of quality. Third, social innovation is to solve social problems by creating new services or products (Cajaiba-Santana, 2014; Wu et al., 2020). In the future, research can focus on products of social innovation and provide evidence for whether or not the consumer price discrimination in minority entrepreneurship exists. Finally, we have only examined the existence of consumer price discrimination in minority entrepreneurship; other discriminations, such as employment discrimination and company valuation discrimination, are still unclear. We hope that future research will complement and expand our study by exploring other phenomena of discrimination in entrepreneurship. Despite these limitations, we believe that our findings challenge the common view of minority entrepreneurship, and hope that both academics and practitioners will benefit from them.

## Conclusion

Economic globalization has promoted political, economic, and cultural exchanges worldwide. Ethnic minority groups have been increasingly involved in multinational economic activities. Prior empirical research shows that minority groups have a great influence in the development and growth of China (Howell, 2019). However, consumer discrimination is a barrier for minority entrepreneurship and a critical obstacle that must be overcome for minority entrepreneurs (Gomolka, 1977). To our knowledge, studies on consumer price discrimination of minority entrepreneurs and its antecedents in China are still rare. Only a few scholars have found that minority-operated enterprises perform worse than Han-operated enterprises, a disparity that suggests discrimination might be at play (He et al., 2019; Howell, 2019). To fill this gap, this paper provides a deep study focusing on two entrepreneurial products (i.e., common products and products with ethnic characteristics) and demonstrates that consumer price discrimination toward the two entrepreneurial products varies extensively. Furthermore, this study shows that prospective consumers expect minority entrepreneurs to offer low prices for common products, while offering high prices and high quality for products with ethnic characteristics. Although this result contrasts somewhat with previous studies, it can be explained by the role of culture in entrepreneurship (Rafiq, 1992; Morrison, 2000; Ma and Todorovic, 2012; Minkov and Hofstede, 2012; Thai and Turkina, 2014).

This study suggests how minority start-ups can take advantage of the price discrimination and strengthen customers’ impression of ethnic brands: consumers assume that minority entrepreneurs sell products with ethnic cultural characteristics at higher prices than common products produced by Han-majority entrepreneurs, and are therefore willing to accept higher prices for those items. This paper contributes to minority entrepreneurship by emphasizing the core role of ethnic culture, fully exploring its cultural connotations, transforming the creativity of cultural characteristics into economic benefits, and ensuring the inheritance and protection of ethnic culture in the process of entrepreneurship. Furthermore, the empirical investigation of ethnic cultural characteristics may drive policymakers to devise better policies to support minority entrepreneurship.

## Data Availability Statement

The datasets presented in this article are not readily available because the dataset were collected through behavioral experiments. Requests to access the datasets should be directed to XL, liao_shane@163.com.

## Ethics Statement

This study was reviewed and approved by the Ethics Committee of Southwest Jiatong University. This study was carried out in accordance with the recommendations of the Ethics Committee of Academic Committee at the Southwest Jiaotong University with informed consent from all participates. All participates gave written informed consent in accordance with the Declaration of Helsinki. The protocol was approved by the Ethics Committee of Academic Committee.

## Author Contributions

FL, XL, and CM participated in the design of this study, performed the statistical analysis, carried out the study, and collected important background information. XL drafted the manuscript. FL, XL, and CM carried out the concepts, design, definition of intellectual content, literature search, data acquisition, data analysis, and manuscript preparation. All authors read and approved the final manuscript.

## Conflict of Interest

The authors declare that the research was conducted in the absence of any commercial or financial relationships that could be construed as a potential conflict of interest.

## Appendix

### Variable Measurement

All variables were measured by asking participants to evaluate the extent to which their perception of entrepreneurial products and individuals.

1 means “disagree strongly” and 7 means “agree strongly.”

#### Perception of Product Quality

The likelihood that the product would be reliable is very high.

The workmanship of product would be very high.

This product should be of very good quality.

The likelihood that this product is dependable is very high.

This product would seem to be very durable.

#### Perception of Founder Quality

From this product perspective, I think the entrepreneur is very:

1. Honest.
2. Dependable.
3. Well-organized.
4. Confident.
5. Likeable.
